# *Helicobacter pylori* eradication in renal transplant candidates

**DOI:** 10.1590/2175-8239-JBN-2021-0097

**Published:** 2022-01-07

**Authors:** Mariana E. Maioli, Raquel F. N. Frange, Cintia M. C. Grion, Vinicius D. A. Delfino

**Affiliations:** 1 Universidade Estadual de Londrina, Departamento de Clínica Médica, Londrina, PR, Brasil.; 2 Pontifícia Universidade Católica, Departamento de Clínica Médica, Londrina, PR, Brasil.

**Keywords:** Renal Insufficiency, Chronic, Helicobacter pylori, Kidney Transplantation, Insuficiência Renal Crônica, Helicobacter pylori, Transplante de Rim

## Abstract

**Introduction:**

Treatment for *Helicobacter pylori (H. pylori)* infection is recommended in transplant candidates due to the association between this infection and gastrointestinal disorders, which could significantly increase morbidity after renal transplantation with the use of immunosuppression. The objective of this study was to analyze the rate of eradication of *H. pylori* after antimicrobial treatment in chronic kidney disease patients who are candidates for kidney transplantation.

**Methods:**

A multicenter prospective cohort study was conducted. All adult chronic kidney disease patients seen at our institution were included. In the pre-transplantation evaluation, 83 patients underwent an upper gastrointestinal endoscopy with 2 diagnostic methods to detect *H. pylori*: histology and the rapid urease test. In total, 33 patients with *H. pylori* infection received treatment with 20 mg omeprazole, 500 mg amoxicillin, and 500 mg clarithromycin once daily for 14 days. Another upper gastrointestinal endoscopy was performed 8 to 12 weeks after the end of treatment to check for healing.

**Results:**

The study showed a prevalence of *H. pylori* in 51 (61.4%) patients. Histology was positive in 50 (98%) patients and the rapid urease test was positive in 31 (60.8%). The infection eradication rate was 48.5% (16 patients).

**Conclusions:**

There was a high prevalence rate of *H. pylori* and a low eradication rate after the long-term antimicrobial triple scheme used. The association of the rapid urease test with gastric mucosa histology did not increase the detection rate of *H. pylori*.

## Resumo

**Introdução:**

Recomenda-se o tratamento da infecção por *Helicobacter pylori* (*H. pylori*) nos candidatos a transplante devido à associação entre esta infecção e distúrbios gastrointestinais, que podem aumentar significativamente a morbidade após transplante renal com o uso de imunossupressão. O objetivo deste estudo foi analisar a taxa de erradicação do *H. pylori* após tratamento antimicrobiano em pacientes com doença renal crônica, candidatos a transplante renal.

**Métodos:**

Realizou-se um estudo de coorte prospectivo multicêntrico. Incluímos todos os pacientes adultos com doença renal crônica atendidos em nossa instituição. Na avaliação pré-transplante, 83 pacientes foram submetidos a uma endoscopia digestiva alta com 2 métodos diagnósticos para detecção do *H. pylori*: histologia e teste rápido de urease. No total, 33 pacientes com infecção por H. pylori receberam tratamento com 20 mg de omeprazol, 500 mg de amoxicilina e 500 mg de claritromicina uma vez ao dia durante 14 dias. Outra endoscopia digestiva alta foi realizada 8 a 12 semanas após o término do tratamento para verificação da resposta.

**Resultados:**

O estudo mostrou prevalência de *H. pylori* em 51 (61,4%) pacientes. A histologia foi positiva em 50 (98%) pacientes e o teste rápido de urease foi positivo em 31 (60,8%). A taxa de erradicação da infecção foi 48,5% (16 pacientes).

**Conclusões:**

Após o esquema triplo antimicrobiano de longo prazo utilizado, houve uma alta taxa de prevalência de *H. pylori* e baixa taxa de erradicação. A associação do teste rápido de urease com a histologia da mucosa gástrica não aumentou a taxa de detecção do *H. pylori*.

## Introduction

*Helicobacter pylori (H. pylori)* is a human pathogen with worldwide distribution^1^, responsible for the most prevalent bacterial infection currently known^2^. It is an etiologic agent of gastrointestinal tract comorbidities varying from mild to severe^3^^,^ 4. The prevalence of *H. pylori* ranges from 30 to 80% in several countries. Despite the high prevalence, clinical manifestations are rare in most patients who carry *H. pylori* in their gastrointestinal tracts, and only a minority of patients develop symptoms^5^.

The study of Homse and colaborators^6^ showed that 100% of these patients present some form of high endoscopic alteration, many of which are potentially severe, including peptic ulcers, gastritis, erosive duodenitis, and gastric intestinal metaplasia^7^. *H. pylori* causes chronic gastric inflammation, which can progress into precancerous alterations such as atrophic gastritis and intestinal metaplasia^8^.

There are currently several methods to diagnose *H. pylori*. Some methods require a prior upper gastrointestinal endoscopy for access to the necessary material^2^. A biopsy of the gastric mucosa is required for some diagnostic methods such as: histology, culture, polymerase chain reaction, and the rapid urease test^2^. Histology is considered the gold standard method for diagnosis of infection by *H. pylori*. It also provides relevant information for the detection of numerous diseases of the esophagus and gastric mucosa^2^. The rapid urease test uses the ability of *H. pylori* to synthesize large amounts of urea as the basis for diagnosis and presents advantages such as low cost, availability, and high specificity, making it widely used in clinical practice.

Treatment for *H. Pylori* infection has been recommended in transplant candidates because of the association between this infection and gastrointestinal disorders such as peptic ulcers, gastric hyperplastic polyps, gastric adenomas, gastric cancers, and mucosa associated-lymphoid-type (MALT) lymphomas^9^^,^ 10 ^,^ 11. With the use of immunosuppression, these disorders could become serious, significantly increasing morbidity after renal transplant. The American College of Gastroenterology Guideline^12^ recommends that clarithromycin triple therapy and bismuth quadruple therapy for *H. pylori* be administered for 14 days, similar to the current recommendations for prolonged treatments (10 to 14 days)^13^^,^ 14.

The goal of this study was to analyze the efficacy of an antimicrobial regime on the eradication of *H. pylori* infection in patients with chronic renal disease, who were candidates for renal transplant.

## Methods

A multicenter prospective cohort study was conducted from May 20^th^, 2016 to November 23^rd^, 2017.

All patients over 18 years of age with chronic kidney disease enrolled in the renal transplant service of the Evangelical Hospital of Londrina and treated in the 6 dialysis clinics in the North of Parana were included in the study. The clinics and locations were as follows: Histocom, Londrina, which treats approximately 200 dialysis patients a month; the Kidney Institute, Londrina, with approximately 65 dialysis patients a month; the Kidney Institute, Cornelio Procopio, with approximately 145 dialysis patients a month; Nefronor, Cornelio Procopio, with approximately 120 dialysis patients a month; and Kidney Institute, Santo Antônio da Platina, with approximately 180 dialysis patients a month. All these units treat patients from the Public or Private Health System who are enrolled in the renal transplant service at the Evangelical Hospital of Londrina.

Patients with the following characteristics were excluded: pregnant, those with a recent history (less than three months) of *H. pylori* infection, abdominal surgery, or high digestive hemorrhage, use of any antibiotic in the past 30 days, and history of allergies to any of the compounds of the therapeutic plan (omeprazole, amoxicillin, or clarithromycin) to be used in the treatment of *H. pylori* infection*.*

On the day of the procedure, the patients were interviewed regarding medications of continuous use and gastrointestinal tract symptoms. Upper gastrointestinal endoscopies were performed in the ambulatory service by a member of the Gastroenterology/Endoscopy service of the Evangelical Hospital of Londrina. Patients on continuous use of gastric protectors were advised to suspend the medication for 7 days before the endoscopy. Patients with *H. pylori* infections were treated daily with 20 mg omeprazole in the morning, in a fasting state, and 500 mg amoxicillin plus 500 mg clarithromycin at night, for 14 days. On hemodialysis days, patients were instructed to take the antibiotic after the sessions.

Data on general characteristics of the studied population were obtained: age, sex, ethnicity, origin, place of residence, and hemodialysis clinic. The following clinical data were collected through an interview: underlying disease, duration of hemodialysis prior to upper gastrointestinal endoscopy, associated comorbidities, previous and/or current smoking history, use of anti-hypertensives, use of gastric protectors, and presence of gastrointestinal tract symptoms in the three months prior to endoscopy. The data sources were registered in the patients' charts in the 6 dialysis units.

The first upper gastrointestinal endoscopy was performed at the time of pre-renal transplantation assessment at the clinic. The rapid urease test and histology, performed through gastric biopsy, were used to confirm *H. pylori* infection. A positive finding in one of these tests was indicative of *H. pylori* infection.

The biopsy was performed in two gastric regions: antrum and body. A third region was assessed in case of inflammation, a suggestive aspect of intestinal metaplasia or neoplasia. The fragments were fixed in 10% formalin and processed for histology and staining with Giemsa. Evaluations were performed by a single pathology laboratory.

Gastritis was classified by the Sidney System (topography: pangastritis, gastritis of body and antrum; category: enantematous, plane erosive and elevated, atrophic, hemorrhagic, reflux, hyperplastic gastric folds; and level of intensity: mild, moderate, severe).

The rapid urease test consisted of immersion of a gastric mucosa fragment of the antrum region into a vial containing urea and phenol red, an indicator of pH (*H. pylori* produces a urease enzyme that turns urea into carbon dioxide and ammonia, leading to an elevation in pH and color alteration to a shade of pink). The test was considered positive when the alteration occurred within two to sixty minutes.

Patients positive for *H. pylori* infection by at least one of the above methods received treatment according to the American College of Gastroenterology Guideline^12^. After one week of treatment, the patients were contacted by telephone to verify adherence to the treatment plan. At this point, the importance of completing treatment was reinforced. After completion of treatment, full compliance was confirmed when patients returned to see a nephrologist.

Eight to 12 weeks after completion treatment, a control upper gastrointestinal endoscopy was performed as the criterion for eradication. The patient was considered negative when both tests were negative. Patients with a negative *H. pylori* test in the first upper gastrointestinal endoscopy did not undergo the second endoscopy.

### Ethical aspects

This research was approved by the National Research Ethics Committee through the Presentation for Ethical Appreciation Certificate nº 54971916.3.0000.5231 and by the Ethics Committee on Research on Human Beings of the State University of Londrina/ North of Parana University Hospital according to report nº 1.565.003281 on the 20^th^ of May 2016. All patients were aware of the nature and goals of the study, agreed to participate, and signed the informed consent term.

### Statistical analysis

The data were analyzed with the Windows' Medcalc program, version 18.0 (Medcalc Software, Ostend, Belgium).

The data are descriptively presented using simple frequencies (relative and absolute), means and deviation rates, or medians and interquartile range (IQR) depending on variable distribution. The data distribution was tested with the Shapiro-Wilk test. The frequencies were described as raw number or percentage, represented in contingency tables, and compared with the Fisher's exact test.

## Results

In total, 83 patients with a median age of 47 years (IQR: 38 - 56) were analyzed. The main underlying diseases were systemic arterial hypertension and diabetes mellitus, while the main dialysis type was hemodialysis. The median duration of dialysis before the upper gastrointestinal endoscopy was 14 months (IQR: 6 - 48). Table 1 presents the demographic and clinical characteristics of pre-transplantation patients.

**Table 1:** Demographic and clinical characteristics of patients in the pre-transplant evaluation

Variables	Absolute frequency (n=83)	Relative frequency (%)
Sex		
Male	43	51.8
Female	40	48.2
Ethnicity/ color		
White	39	46.9
Black	30	36.1
Brown	8	9.6
Asian	6	7.2
Basal Disease		
SAH	36	43.3
DM	22	26.5
SAH and DM	17	20.4
Glomerulonephritis	5	6.0
Other etiologies	3	3.6
HD Modality	82	98.7
Current Smoker	26	31.3

SAH = Systemic Arterial Hypertension; DM= Diabetes Mellitus; HD= Hemodialysis.

**Figure 1:**
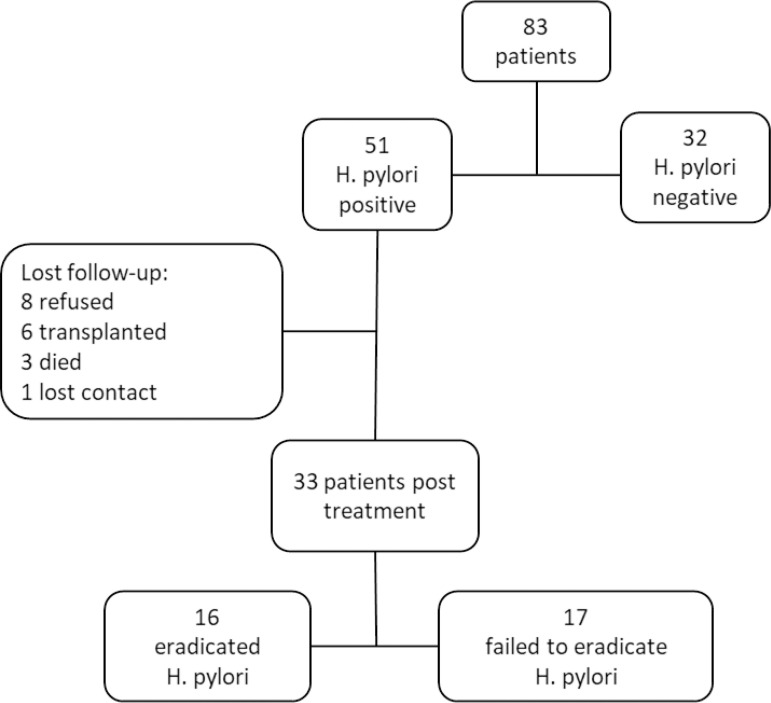
Flowchart of patients in the study.

The study showed a 61.4% prevalence of *H. pylori*. Histology demonstrated 98% positivity as a diagnostic method for *H. pylori* and the rapid urease test, 60.8%. The rapid urease test detected *H. pylori* separately in only one patient. Of the 51 patients who tested positive for *H. pylori*, 18 were lost to follow up. Thus, 33 patients were part of the treatment protocol. The infection eradication rate was 48.5% (Figure1).

Findings were positive in 96.4% of endoscopies. The most commonly found lesion was enanthematous pangastritis. Few patients had ulcers. Only one patient presented a precancerous lesion, which was Barrett's esophagus. No malignant lesions were detected. There were no associations between the endoscopic findings, symptomatology, and the presence of *H. pylori* based on the Fisher's exact test (Table 2).

**Table 2:** Initial endoscopic findings in asymptomatic and symptomatic patients (n=83)

Endoscopic Findings (%)	SYMPTOMATIC		ASYMPTOMATIC	
	*H. pylori* (+)	*H. pylori* (-)	*H. pylori* (+)	*H. pylori* (-)
Erosive Esophagitis (27.7)	10	5	2	6
Non-erosive Esophagitis (13.2)	5	1	3	2
Hiatal Hernia (13.2)	4	3	0	4
Active GU (1.2)	1	0	0	0
Erosive Gastritis (6)	1	1	0	3
Enan Gastritis (8.4)	3	1	0	3
Enan Pangastritis (83)	29	14	18	8
Erosive Duodenitis (24)	6	6	4	4
Enan Duodenitis (36)	14	6	6	4
Barret´s Esophagus (2.4)	1	0	1	0
Active UDuo (1.2)	1	0	0	0
Healing UDuo (1.2)	1	0	0	0
Healed UDuo (2.4)	0	0	2	0

*H. pylori* (+): Positive for *Helicobacter pylori; H. pylori* (-): Negative for *Helicobacter pylori*, GU= Gastric ulcer;Enan= Enantematic; UDuo= Duodenal Ulcer.

Gastrointestinal symptoms were reported by 61.4% of patients. Epigastric pain did not occur in patients with endoscopic findings of ulcer. Nausea and pyrosis were frequent symptoms. There was a tendency for vomiting to be more associated to enantematous pangastritis (Table 3).

**Table 3:** Gastrointestinal symptomatology and endoscopic findings in patients in the pre-transplant evaluation

	Epigastric Pain N (%)	Pirosis N (%)	Regurgitation N (%)	Fullness N (%)	Satiety N (%)	Eructation N (%)	Abdominal Distension N (%)	Nausea N (%)	Vomit N (%)	Anorexia N (%)
Erosive Esophagitis (n=15)	5(31)	8(50)	2(12)	5(31)	3(19)	5(31)	3(19)	8(50)	2(12)	1(6)
Non-erosive Esophagtis (n=6)	1(17)	4(67)	0	0	0	0	1(17)	5(83)	1(17)	1(17)
Hiatal Hernia (n=7)	3(43)	2(28)	3(43)	3(43)	3(43)	0	3(43)	4(57)	1(14)	1(14)
Active GU (n=1)	0	1(100)	0	1(100)	0	0	0	0	0	0
Erosive Gastritis (n=2)	0	0	0	2(100)	0	2(100)	0	2(100)	2(100)	0
Enan Gastritis (n=4)	1(25)	2(50)	2(50)	2(50)	0	1(25)	0	1(25)	1(25)	0
Erosive Pangastritis (n=1)	0	1(100)	0	0	0	0	0	0	0	0
Enan Pangastritis (n=43)	12(28)	23(43)	12(28)	11(25)	9(21)	9(21)	9(21)	25(58)	6(14)	4(9)
Erosive Duodenitis (n=12)	3(25)	4(33)	6(50)	4(33)	4(33)	3(25)	2(17)	5(42)	1(8)	0
Enan Duodenitis (n=20)	8(40)	11(55)	4(20)	6(30)	4(20)	3(15)	6(30)	12(60)	3(15)	1(5)
Barret’s Esophagus (n=1)	0	0	1(100)	1(100)	1(100)	0	0	0	0	0
Active UDuo (n=1)	0	1(100)	0	0	0	0	0	0	0	0
Healed UDuo (n=1)	0	0	0	1(100)	1(100)	0	1(100)	1(100)	0	0

Legends: GU = Gastric Ulcer; Enan = Enantematic; UDuo = Duodenal Ulcer; UDuo = Duodenal Ulcer.

After 7 days of follow up by telephone contact, all patients presented good treatment compliance. Complete compliance was confirmed when patients returned to a nephrologist after 14 days of treatment; treatment did not include supervised pill-taking.

## Discussion

A high prevalence of *H. pylori* was found in renal transplant candidates. The association of diagnostic methods was disadvantageous to the detection of *H. pylori* by upper gastrointestinal endoscopy. The eradication rate after using the triple scheme for 14 days was low in the included patients.

The eradication rate of *H. pylori* in the general population after treatment with the first line triple scheme (proton pump inhibitor, amoxicillin, and clarithromycin) has decreased in recent years, especially after the use of the shorter scheme (7 days)^15^.It was 93.5% in 2003 and by 2012, it had reduced to 78.8%^15^. Some meta-analyses show higher eradication rates with a longer treatment of 14 days^16^^,^ 17 ^,^ 18 ^,^ 19. Recent literature reviews demonstrated that the increase in triple therapy duration increased the *H. pylori* eradication rate (72.9 vs. 81.9%), independently of the type and dosage of antimicrobials^17^.

The eradication rate of *H. pylori* in chronic kidney disease patients varies according to the antimicrobial schemes used, whether triple, quadruple, or sequential. In the Seyyedand and collaborators study, the triple scheme with 30 mg lansoprazole twice a day, 250 mg clarithromycin twice a day, and 500 mg amoxicillin twice a day for 14 days, presented a 76.7% eradication rate for *H. pylori* 20 *.* Another double blind prospective clinical study compared two groups of chronic kidney disease patients. One group of 35 patients received the full dosage of the scheme with 20 mg omeprazole, 500 mg clarithromycin, and 1000 mg amoxicillin twice a day for 14 days, while the other group of 31 patients received the same drugs throughout the same period, but once a day. The eradication rate of both groups was 73% with no statistical difference between the two regimes (p= 0.973)^21^.

Although the extended time frame of the triple treatment was preferred by our patients, we found a lower eradication rate. This could have been due to poor compliance to the prolonged treatment or an unknown resistance to one of the medications used, especially clarithromycin. Resistance to clarithromycin is still the most common cause of triple therapy failure, and the period of treatment does not affect the high rates of clarithromycin resistance in the general population^13^. In a previous study that evaluated the 5 macro-regions of Brazil, bacterial resistance to clarithromycin varied from 15 to 20%^22^. The current study could not assess resistance to clarithromycin, since neither a culture, nor an antibiogram were conducted^13^.

The influence of pre-therapeutic histological parameters on *H. pylori* eradication rate is still controversial. Georgopoulos and collaborators suggested that the coexistence of high scores of antral gastritis degree and activity with any degree of corpus gastritis may favorably affect the outcome of treatment, supporting the idea of facilitated diffusion of antibiotics in the inflamed mucosa^23^. However, in the current work, we found a lower eradication rate, although the most prevalent endoscopic finding was pangastritis and enema.

*H. pylori* is the most important etiological factor for gastric cancer. *H. pylori* causes chronic gastric inflammation that can progress to precancerous alterations in atrophic gastritis and in intestinal metaplasia. The risk of gastric cancer increases according to the extension and severity of these precancerous alterations. Eradication of *H. pylori* can induce resolution of gastric inflammation, stop progression of gastric mucosa damage, prevent additional damage to the DNA induced by *H. pylori*, improve gastric acid secretion, and restore normality of the internal environment^8^. Thus, we believe that treatment with an antimicrobial regime should be considered in patients with *H. pylori* and chronic kidney disease.

Patients with chronic kidney disease present a greater risk of gastroduodenal disorders. It is recommended that all hemodialysis and peritoneal dialysis patients be submitted to endoscopic evaluation to reduce the chances of developing peptic ulcers, especially in patients with a history of gastroduodenal bleeding or use of anticoagulants and/or non-steroidal anti-inflammatory drugs^24^. We used two diagnostic methods associated with upper gastrointestinal endoscopy to increase the probability of detection of *H. pylori*. The literature demonstrates high sensitivity and specificity of the rapid urease test, varying between 80 and 100% and 97 and 99%, respectively^16^^,^ 25. However, in the current study, *H. pylori* detection by the rapid urease test was low. A disadvantage of the test that could explain this situation is false-negative results due to the reduction in the activity of urease caused by recent use of antimicrobials, bismuth compounds, or PBIs, or because of achlorhydria. In addition, the presence of gastric bleeding from uremia reduces the sensitivity and specificity of the method. Furthermore, the amount of bacteria present affects the sensitivity of the test; quantities above 10,000 in the sample indicate positive results, while quantities below this can generate false negatives. The rapid urease test should be performed with a biopsy of one gastric region (antrum), differently from the histology from biopsy of two gastric regions (body and antrum). For now, we suggest that the association of both methods in our patients increased the evaluation costs and did not lead to an increase in detection rate.

Patients with chronic kidney disease have a high incidence of gastrointestinal diseases, even though the occurrence and type of symptoms can vary considerably between patients^11^. Many gastrointestinal symptoms such as anorexia, nausea, vomiting, and dyspepsia are common in patients with chronic kidney disease waiting for a renal transplant. These symptoms could be indicative of uremia or the result of medications and electrolyte imbalance, which makes it difficult to confidently predict the presence of a meaningful lesion in the superior gastrointestinal tract. However, many chronic kidney disease patients with peptic ulcers are asymptomatic^26^^,^ 27 and can present significant complications before and after renal transplant, especially during the period of high immunosuppression^28^. This finding is very relevant, as an active peptic ulcer is a contraindication for renal transplant^29^ and strongly supports the recommendation for upper gastrointestinal endoscopies before transplantation. There is no solid evidence on the role of routine triage with upper gastrointestinal endoscopy for *H. pylori* in asymptomatic candidates during evaluation before renal transplantation and no consensus between transplant centers^30^. In a recent study performed in the same renal transplant center, Homse and collaborators showed that lesions in the gastrointestinal tract were found in all analyzed patients, even though patients did not present symptoms^6^. It is believed that the findings of the study justify the recommendation for an upper gastrointestinal endoscopy in the preparation routine for renal transplantation in chronic kidney disease patients.

### Implications, strengths and limitations

This is one of the first studies to evaluate findings from upper gastrointestinal endoscopies for pre-transplant preparation and the efficiency of *H. pylori* treatment in renal transplant candidates. The verification of drug compliance by phone contact 7 days after the beginning of treatment was also considered a strength of the study. Complete compliance was confirmed when patients were seen by the nephrologist after the end of treatment, but supervised pill-taking was not performed. An association was found between *H. pylori* infection and upper gastrointestinal endoscopy findings in this population. Some limitations of the study should be considered, such as the low number of patients analyzed and the losses to follow up, which were partly due to the difficulties faced by patients to access transplant centers to repeat the upper gastrointestinal endoscopy. Therefore, our results should be interpreted with caution. Another limitation of the study was that no upper digestive endoscopy was performed after kidney transplantation. This was not included in the original study, as the coverage of its performance in asymptomatic patients is limited in the National Health System. Another limitation was that two of the drugs used against *H. pylori* are filtered during hemodialysis (amoxicillin and clarithromycin). To minimize this effect, patients were instructed to take these medications after hemodialysis.

There is no clinical protocol for a detailed endoscopic evaluation and eradication therapy for *H. pylori* in patients on dialysis. Therefore, future studies must be developed to confirm and expand the findings of the current study^24^^,^ 31.

## Conclusion

A high prevalence of *H. pylori* was found in renal transplant candidates, and triple antimicrobial therapy applied over a long period had a low eradication rate. The performance of routine upper gastrointestinal endoscopy in pre-transplant evaluation detected gastrointestinal lesions in most patients and the endoscopic findings did not relate to symptoms. The association of the rapid urease test with gastric mucosa histology did not increase the detection rate of *H. pylori*.
